# Supplementary material for: Co-Loading of Black Phosphorus Nanoflakes and Doxorubicin in Lysolipid Temperature-Sensitive Liposomes for Combination Therapy in Prostate Cancer
Source: Int J Mol Sci. 2023 Dec 21;25(1):115. doi: 10.3390/ijms25010115 (PMC10779057; doi:10.3390/ijms25010115)
Supplement: Supplementary file 1 [file ijms-25-00115-s001.zip › ijms-2711275-supplementary.pdf]

Co-Loading of Black Phosphorus Nanoflakes and Doxorubicin in Lysolipid Temperature-Sensitive Liposomes for Combination Therapy in Prostate Cancer

Chandrima Das [1], Cristina Martín [2], Sebastian Habermann [3], Harriet Rose Walker [1], Javed Iqbal [1], Jacobo Elies [1], Huw Simon Jones [1], Giacomo Reina [3,*] and Amalia Ruiz [1,*]

1 Institute of Cancer Therapeutics, School of Pharmacy and Medical Sciences, Faculty of Life Sciences, University of Bradford, Bradford BD7 1DP, UK; c.daschandrima@gmail.com (C.D.); h.r.walker@bradford.ac.uk (H.R.W.); javediqbal.georgian@gmail.com (J.I.); j.eliesgomez@bradford.ac.uk (J.E.); h.s.jones@bradford.ac.uk (H.S.J.)
2 Department of Bioengineering, Universidad Carlos III de Madrid, 28911 Leganés, Spain; cristima@ing.uc3m.es
3 Empa Swiss Federal Laboratories for Materials Science and Technology, Lerchenfeldstrasse 5, 9014 St. Gallen, Switzerland; sebastian.habermann@empa.ch
* Correspondence: giacomo.reina@empa.ch (G.R.); g.ruizestrada@bradford.ac.uk (A.R.)

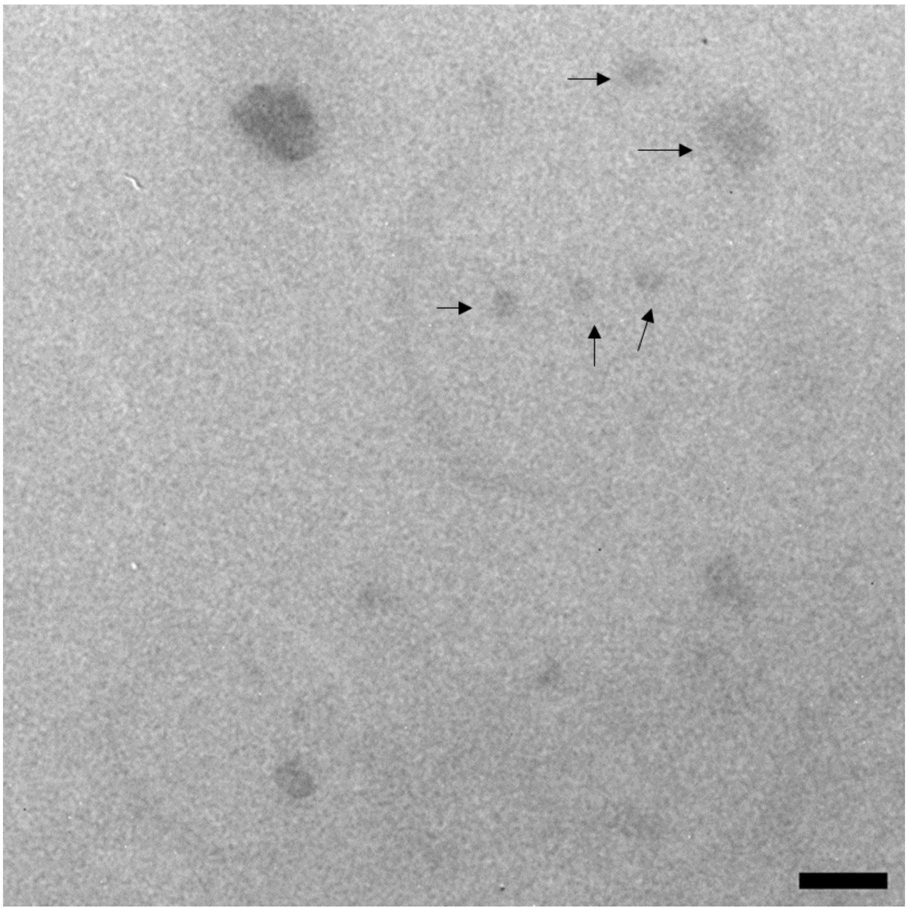

Figure S1. TEM micrograph of LTSL-BPNF, primary flakes loaded in the liposomes are below 20 nm. Scale bar 50 nm.

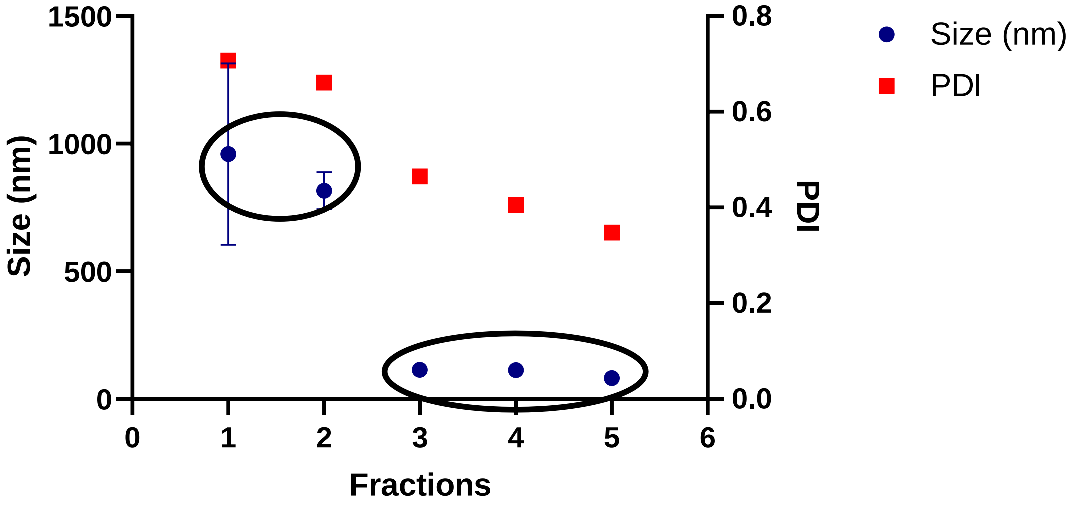

Figure S2. Colloidal properties of the eluted fractions of LTSL-BPNF after purification using a PD10 column.
